# A Online NIR Sensor for the Pilot-Scale Extraction Process in Fructus Aurantii Coupled with Single and Ensemble Methods

**DOI:** 10.3390/s150408749

**Published:** 2015-04-14

**Authors:** Xiaoning Pan, Yang Li, Zhisheng Wu, Qiao Zhang, Zhou Zheng, Xinyuan Shi, Yanjiang Qiao

**Affiliations:** College of Chinese Medicine, Beijing University of Chinese Medicine, South of Wangjing Middle Ring Road, Chaoyang District, Beijing 100102, China; E-Mails: panxiaoning1112@163.com (X.P.); limingyangsoul@163.com (Y.L.); zhangqiao0824@foxmail.com (Q.Z.); saturday1226@163.com (Z.Z.); shixinyuan01@163.com (X.S.)

**Keywords:** online NIR sensor, PLS, bagging-PLS, *Fructus aurantii*, synergy interval partial least squares

## Abstract

Model performance of the partial least squares method (PLS) alone and bagging-PLS was investigated in online near-infrared (NIR) sensor monitoring of pilot-scale extraction process in *Fructus aurantii*. High-performance liquid chromatography (HPLC) was used as a reference method to identify the active pharmaceutical ingredients: naringin, hesperidin and neohesperidin. Several preprocessing methods and synergy interval partial least squares (SiPLS) and moving window partial least squares (MWPLS) variable selection methods were compared. Single quantification models (PLS) and ensemble methods combined with partial least squares (bagging-PLS) were developed for quantitative analysis of naringin, hesperidin and neohesperidin. SiPLS was compared to SiPLS combined with bagging-PLS. Final results showed the root mean square error of prediction (RMSEP) of bagging-PLS to be lower than that of PLS regression alone. For this reason, an ensemble method of online NIR sensor is here proposed as a means of monitoring the pilot-scale extraction process in *Fructus aurantii*, which may also constitute a suitable strategy for online NIR monitoring of CHM.

## 1. Introduction

In 2004, the U.S. Food and Drug Administration issued its “Process Analytical Technology (PAT) Industry Guide,” which encourages pharmaceutical companies to develop innovative drugs and ensure quality during manufacturing [1]. PAT is considered a quality system. Its purpose is to collect real-time information on all aspects of critical processes and to guide the process towards its desired state, hence ensuring the quality of the final product. Online technology is mentioned several times in the guide. Online near-infrared (NIR) sensors have been proven to be one of most efficient and advanced tools available for monitoring and controlling the production and processing of food, agricultural products, pharmaceuticals and petroleum. Killner *et al*. utilized a NIR sensor for online monitoring of the progress of the catalyzed transesterification reactions of soybean oil that produced biodiesel [2]. Collell *et al*. used three different NIR sensors to predict superficial water activity and moisture content in two types of fermented sausages [3]. Marín-González *et al*. evaluated the performance and accuracy of online measurement of soil properties using indirect spectral response in NIR spectral range [4]. All these investigations mentioned above confirmed that online NIR sensors can be used to monitor and control the quality of production and processing, but all used a single quantification PLS model.

NIR has recently come to be regarded as an excellent sensor for the monitoring of processes in Chinese Herbal Medicine (CHM). Online NIR applications have been reported in CHM. The use of online NIR sensors for quality control reveals an increasing trend. Firstly, online NIR sensor can record spectra for liquid CHM samples, e.g., the extraction solution of extraction processes, concentration processes, alcohol precipitation processes and purification processes. Wu *et al*. monitored the extraction process of chlorogenic acid from *Lonicera japonica* using online NIR sensors and established multivariate models including PLS and interval partial least squares (iPLS) models, which produced encouraging results regarding the reliability of online NIR sensors in the monitoring of extraction processes in CHM [5]. Qu *et al*. showed that the process of concentrating the alcohol extract from red ginseng alcohol extraction can be monitored online using a NIR sensor coupled with a PLS regression model [6]. Jin *et al*. investigated the use of a NIR sensor combined with particle swarm optimization-based (PSO-based) least square support vector machine (LS-SVM) regression and PLS regression for quantitative online monitoring of alcohol precipitation of the Danhong injection formulation [7]. Liu *et al*. reported that NIR sensors using PLS were used to monitor online the column separation and purification of madecassoside and asiaticoside of *Centella asiatica L. Urban* [8].

Online NIR sensors can record spectra for solid CHM samples, such as tablets, capsules, plasters, and pills. Wan *et al*. developed a NIR sensor and PLS for rapid, nondestructive analysis of the content and moisture levels of semi-manufactured products generated from the preparation of granular dried of gingko leaf dispersible tablets [9]. Geng *et al*. established a method for online analysis of the paeoniflorin content of the Chuanhong Huoxue capsule extraction process with a NIR sensor [10]. Jiang *et al*. investigated the use of NIR combined with PLS regression for online monitoring of the content of baicalin in Shang Jie plaster extract solutions [11]. Jin *et al*. established a simple and speedy method of monitoring the bleeding of Zhongsheng pill powder online based on diffuse reflectance NIR spectra, and the moving block standard deviation (MBSD) method was used to identify the endpoint of the bleeding process [12].

CHMs have their own characteristics, including a complex chemical composition and low-concentration active pharmaceutical ingredients (API). For this reason, any NIR model used to quantify API should be validated. PLS is a popular multivariate calibration technique for quantitative analysis of NIR spectral data [13]. It is a dimension reduction technique that involves finding a set of latent variables of two variable blocks. Although it is very useful in the resolution of calibration problems, the PLS model is susceptible to unrelated and collinear spectral variables [14]. Recently, increasing amounts of attention have been paid to the use of ensemble methods, such as bagging and partial least squares regression (bagging-PLS), in multivariate regression [15]. Bagging was proposed by Breiman. It involves reducing the variation in predictors by aggregating several models obtained in the course of sampling. Bagging involves combining the results of these models into one. In this way, this method can be viewed as combinations of many models and can be used to summarize them. This gives better predictive performance than a single model. It can also prevent over fitting [16]. The robustness of these PLSR models and their predictions can be improved by combining them with ensemble techniques. For example, Viscarra Rossel tested the implementation of bagging with PLSR using vis-NIR and mid-IR diffuse reflectance spectra to predict soil organic carbon, which showed bagging-PLSR to be more robust than PLSR alone [17].

*Fructus aurantii* (Zhiqiao), the dried, immature fruit of *Citrus aurantium* L., is well-known in CHM [18]. Flavonoids are derived from *Fructus aurantii* and via previous modern pharmacological studies and clinical trials they have been proven to have anti-oxidative, anti-inflammatory, antiviral, and anti-dyspeptic effects [19,20,21,22,23].

According to the literature, studies investigating the use of bagging with multivariate calibration of online NIR CHM sensors have only rarely been reported. The purpose of the present paper is to compare the performance of PLS to that of bagging-PLS using online NIR sensors regarding the monitoring of the pilot-scale *Fructus aurantii* extraction process.

## 2. Materials and Methods

### 2.1. Materials

*Fructus aurantii* was purchased from Ben Cao Fang Yuan (Beijing, China). Naringin reference standard (No. 110722-201312), hesperidin reference standard (No. 110721-201316), and neohesperidin reference standard (No. 111857-201102) were supplied by the National Institutes for Food and Drug Control (Beijing, China). Acetonitrile (Fisher Scientific, Fair Lawn, NJ, USA) was HPLC grade. Acetic acid (Beijing Chemical Works, Beijing, China) was analytical grade. Deionized water was prepared by a Milli-Q water system (Millipore Corp., Bedford, MA, USA).

### 2.2. Preparation of Samples

*Fructus aurantii* (6.5 kg) was extracted three times with 10-fold deionized water in a multi-functional extractor (100 L), once every 1.5 h. The speed of the stirring paddle was set to 50 rpm. During the extraction process, the NIR spectra were scanned periodically. According to the content of three ingredients, a reasonable sampling interval was designed (Table 1). During the initial heating and boiling phase, the levels of components varied rapidly, so the sampling interval was set very small. In the second and third stages of the extraction process, the levels of components varied less than during the first extraction stage. In this way, the interval could be adjusted to reduce the amount of work required.

**Table 1:** The sampling interval of extraction process.

Extraction	Extraction Time		
process	Heating	0–1 h	1–1.5 h
1st extraction	3 min	4 min	4 min
2nd extraction	5 min	5 min	5 min
3rd extraction	5 min	6 min	10 min

**Figure 1:**
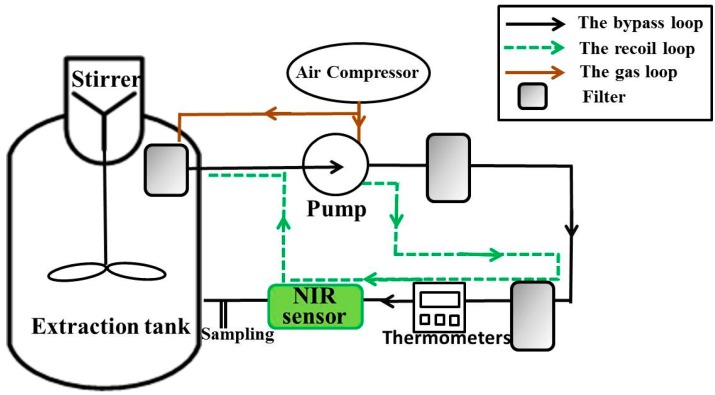
Platform of extraction.

The process system included the online NIR scanning sensor and extraction equipment containing a sampling device (Figure 1). The whole process can be described as follows: the tank was added with CHM and extracted with deionized water. The extraction solution was circulated in the bypass under the action of a pump. Bubbles and solid content could interfere with the spectra to a considerable extent. For this reason, 80 μm and 100 μm filters were used to eliminate these interference factors when the extraction solution passed through the filters during bypass [24].

Temperature was recorded in real time using thermometers. Throughout the extraction process, spectra were collected using online NIR instruments with optical fibers. After the cessation of NIR scanning, the switch was opened, and about 10 mL of extraction solution was collected for HPLC determination.

### 2.3. NIR Equipment and Measurement

The NIR spectra were collected online using fiber optic probes. NIR radiation was applied through a 2 mm optical using an XDS process analyzer and VISION software (Foss NIR System, Foss, Silver Spring, MD, USA). The wavelength range of spectra was between 800 nm and 2200 nm. Each spectrum was the average of 32 scans, and the wavelength increment was 0.5 nm.

### 2.4. HPLC Method for Fructus aurantii

Amounts of naringin, hesperidin and neohesperidin standards were accurately weighed using an XS205DU electronic balance (Mettler Toledo, Greifensee, Switzerland). The samples were dissolved in methanol, and the concentrations of hesperidin, naringin and neohesperidin were 0.1392 mg/mL, 0.1380 mg/mL and 0.1044 mg/mL, respectively. An HPLC assay was used to determine the levels of hesperidin, naringin and neohesperidin [19].

The concentration of the solution obtained from the extraction process was too high for the HPLC assay. For this reason, at the initial heating and boiling phase, 5 mL and 1 mL of the extraction solution were transferred into a 25 mL volumetric flask, and diluted to volume with 20% aqueous methanol, respectively. Meanwhile, during the second heating and boiling phase of extraction process, 2.5 mL and 2 mL of the extraction solution were transferred into 25 mL volumetric flask and diluted to volume with 20% aqueous methanol. During the third extraction stage, the solution was used immediately. The solutions obtained as above were filtered through a 0.45 μm membrane filter for subsequent analysis.

The chromatographic analysis of *Fructus aurantii* was carried out using a Waters 2695 HPLC system and Waters 2996 DAD detector (Waters Technologies, Milford, MA, USA). The sample solutions were analyzed using reverse-phase chromatography on Diamonsil C18 column (250 mm × 4.6 μm, Dikma, Beijing, China) with gradient elution of the mobile phase consisting of acetonitrile and deionized water with 0.1 acetic acid (v/v) at a flow rate of 1.0 mL/min. The column temperature was 30 °C and detection wavelength was set to 283 nm. A 10 μL volume of the extracted fluid was injected into the HPLC system for analysis.

### 2.5. Preprocessing and Variable Selection Methods

To improve the accuracy of the model performance, derivatives, including first (1D) and second derivatives (2D), were used to reduce baseline variation and to enhance spectral features [25]. Then the Savitzky-Golay smoothing filter was combined with 11 points to depress the background noise that had been amplified by the derivative [26]. Standard normal variation (SNV) and multiplicative scatter correction (MSC) were used to reduce the influence of small particles in extraction solution [27,28]. Normalize was also used to preprocess the raw spectra and so produce an accurate model [29].

Chemometric methods of synergy interval partial least squares (SiPLS) and moving window partial least squares (MWPLS) were used to select variables [30,31]. The principle underlying the SiPLS algorithm was that the full spectrum was split into a number of smaller intervals. Several intervals were combined to form a joint model, and these joint models were optimized using the RMSECV value. Several good joint models were combined to select the best sub-interval combination. MWPLS was also used to select variables. The function of the MWPLS model was briefly described and used to identify the informative regions and to approximate latent factors. In effect, a window of size *H* was moved across the data set to collect modeling information. The RMSECV value was calculated and used to find the best spectral regions of size *H*. If the model was of sufficient quality, the value of RMSECV was lower than the value of PLS. In this way, informative regions were optimized so that they had lower RMSECV values than PLS models.

### 2.6. Software and Data Analysis

Data analysis was performed using the Unscrambler 9.6 software package (CAMO Software AS, Trondheim, Norway), and home-made routines programmed in MATLAB code (MATLAB v7.0, the Math Works, Natick, MA, USA). The toolbox of SiPLS and MWPLS that had been used to select the informative variables were downloaded from the Internet [32]. Others algorithms were modified on the basis of Norgaard algorithms developed by the current team. According to Kennard-Stone (KS) algorithm, 75 samples were divided to 50 calibration samples and 25 validation samples. In addition, the coefficient of determination in calibration (R^2^_cal_), the coefficient of determination in cross validation (R^2^_val_), the coefficient of determination in prediction (R^2^_pre_), root mean square error of calibration (RMSEC), root mean square error of cross-validation (RMSECV) and root mean square error of prediction (RMSEP) were used to evaluate the PLS model and SiPLS model. The MWPLS model was evaluated according to the RMSECV value, and the bagging-PLS model was evaluated according to the RMSECV value.

## 3. Results and Discussion

### 3.1. NIR Spectrum Characteristics of Fructus aurantii

**Figure 2:**
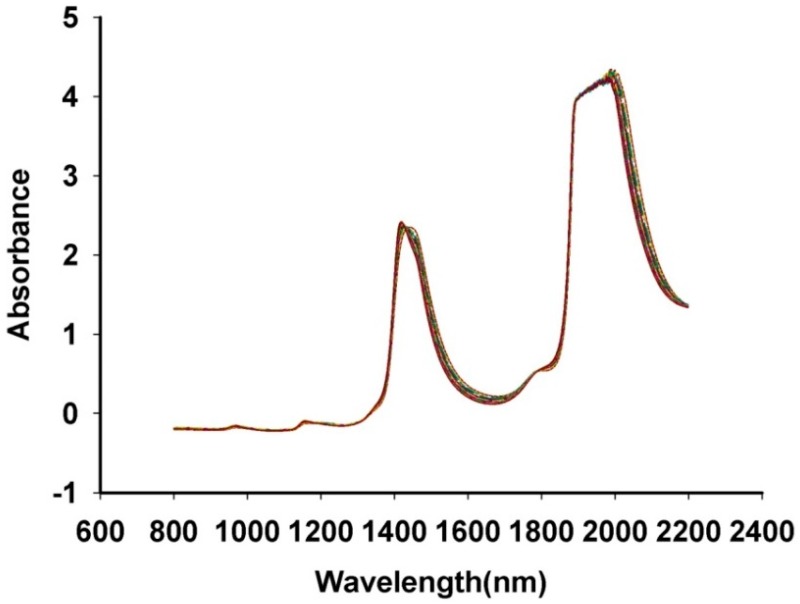
The NIR spectra of *Fructus aurantii*.

The raw spectra are shown in Figure 2. As shown, there was a large fluctuation from 2000 to 2200 nm [33]. Besides, the aqueous solution main absorbance was at about 1950 nm [34]. There were large signal fluctuations in the 780–2100 nm spectral region. Therefore, 800–2200 nm was all used for analysis.

### 3.2. Quantitative analysis of Naringin, Hesperidin and Neohesperidin by HPLC Method

**Figure 3:**
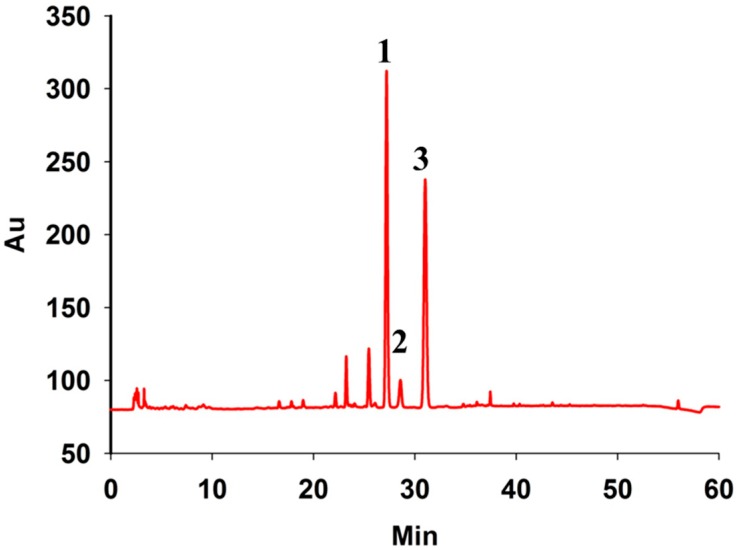
The HPLC chromatograms of *Fructus aurantii.* Peaks: naringin (1); neohesperidin (2); hesperidin (3).

The HPLC chromatograms of the sample are shown in Figure 3. The reference values of three compounds are shown in Table 2. For hesperidin, naringin and neohesperidin, 2 μL, 5 μL, 10 μL, 15 μL, 20 μL and 25 μL standard solutions were injected into the HPLC for analysis. The calibration curves showed good linearity (R^2^ = 0.9991, R^2^ = 0.9999, R^2^ = 0.9996) within line ranges of 0.02784 to 0.348 μg, 0.0276 to 0.345 μg and 0.02088 to 0.261 μg. The precision, repeatability and stability met the requirements of analysis.

**Table 2:** The reference values of the three compounds in *Fructus aurantii*.

Compound	Minimum Value (mg/mL)	Maximum Value (mg/mL)	Average Value (mg/mL)
Hesperidin	0.0146	0.1889	0.0750
Naringin	0.2303	2.5504	1.0002
Neohesperidin	0.1893	2.0272	0.7814

### 3.3. NIR Optimum Result of Preprocessing Methods and Latent Factors

**Figure 4:**
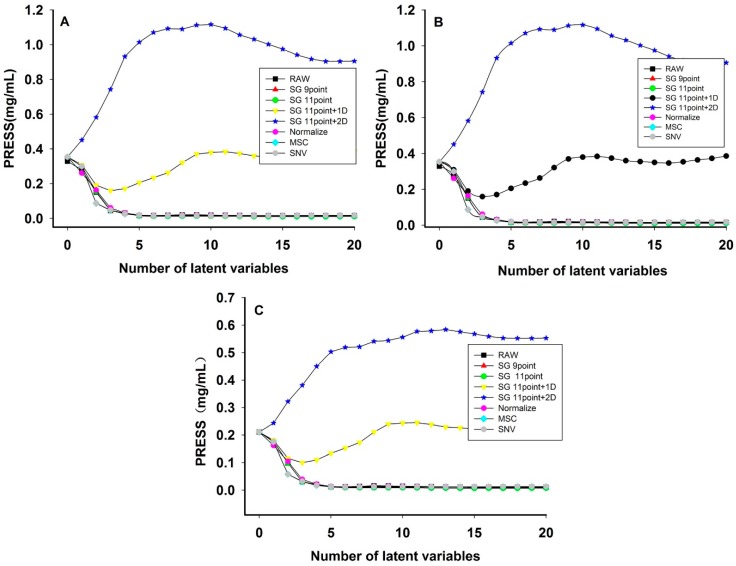
PRESS plot of PLS model using different preprocessing methods of hesperidin (**A**); naringin (**B**); neohesperidin (**C**) (y-axis units added for PRESS).

Savitzky-Golay smoothing (S-G), S-G and first derivative (SG + 1D), S-G and second derivative (SG + 2D), normalize, MSC, and SNV have been used to preprocess the raw spectra. Leave-one-out cross-validation was used to select an appropriate preprocessing method. The number of latent variable factors was investigated. The optimum number of latent factors was determined using the lowest predicted residual sum of squares (PRESS) value (Figure 4).

Figure 4 shows the relationship between latent variable and PRESS in different preprocessing methods. Table 3 shows the result of PLS model with different preprocessing methods. The raw spectra with no preprocessing had the lowest PRESS value, the coefficient of determination (R^2^) closest to 1, and the smallest RMSEC, RMSECV and RMSEP. The results showed the raw spectra were the best method of establishing the PLS model in each quality parameter.

**Table 3:** The results of the PLS model using different preprocessing methods.

Quality Parameter	Preprocessing Methods	Latent Factors	Calibration Set		Validation Set		Prediction Set	
			RMSEC *	R^2^	RMSECV *	R^2^	RMSEP *	R^2^
Hesperidin	RAW	5	0.0067	0.9760	0.0096	0.9530	0.0158	0.9237
	SG9	5	0.0073	0.9721	0.0096	0.9531	0.0159	0.9222
	SG11	5	0.0074	0.9710	0.0096	0.9528	0.0160	0.9217
	SG11 + 1D	3	0.0155	0.8724	0.0274	0.6195	0.0349	0.6258
	SG11 + 2D	1	0.0339	0.3928	0.0496	0.2487	0.0665	0.3582
	Normalize	5	0.0059	0.9817	0.0095	0.9541	0.0156	0.9255
	MSC	5	0.0051	0.9863	0.0091	0.9583	0.0166	0.9119
	SNV	5	0.0052	0.9857	0.0090	0.9585	0.0160	0.9209
Naringin	RAW	5	0.0892	0.9766	0.1334	0.9499	0.1551	0.9544
	SG9	5	0.0916	0.9753	0.1234	0.9569	0.1567	0.9535
	SG11	5	0.0922	0.9750	0.1222	0.9578	0.1568	0.9534
	SG11 + 1D	3	0.2323	0.8411	0.4003	0.5469	0.4342	0.6424
	SG11 + 2D	1	0.4991	0.2665	0.6717	0.2757	0.8544	0.3845
	Normalize	5	0.0854	0.9785	0.1395	0.9450	0.1688	0.9460
	MSC	5	0.0721	0.9847	0.1347	0.9487	0.1738	0.9427
	SNV	5	0.0744	0.9837	0.1349	0.9486	0.1758	0.9414
Neohesperidin	RAW	5	0.0774	0.9705	0.1108	0.9420	0.1197	0.9545
	SG9	5	0.0796	0.9688	0.1062	0.9467	0.1201	0.9542
	SG11	5	0.0801	0.9684	0.1053	0.9476	0.1200	0.9543
	SG11+1D	3	0.1848	0.8318	0.3164	0.5264	0.3386	0.6356
	SG11+2D	1	0.3635	0.3495	0.4943	0.1556	0.6671	0.4145
	Normalize	5	0.0724	0.9742	0.1167	0.9356	0.1288	0.9472
	MSC	5	0.0604	0.9821	0.1116	0.9411	0.2240	0.8405
	SNV	5	0.0622	0.9810	0.1120	0.9407	0.1330	0.9438

* The unit of RMSEC, RMSECV and RMSEP was mg/mL.

### 3.4. Different Variable Selection for PLS Models of Three Compounds in Fructus aurantii

Several thousands of variables were measured in NIR spectra, but the absorption bands overlapped heavily in the NIR region. Some of the variables might not affect the chemical properties of the whole. In order to construct a good calibration model, a method for the extraction of sample-specific and component-specific information from NIR spectra, such as SiPLS and MWPLS should be used. Each optimal SiPLS model was built using a combination of subinterval number 3 and also 20 equidistant subintervals.

**Figure 5:**
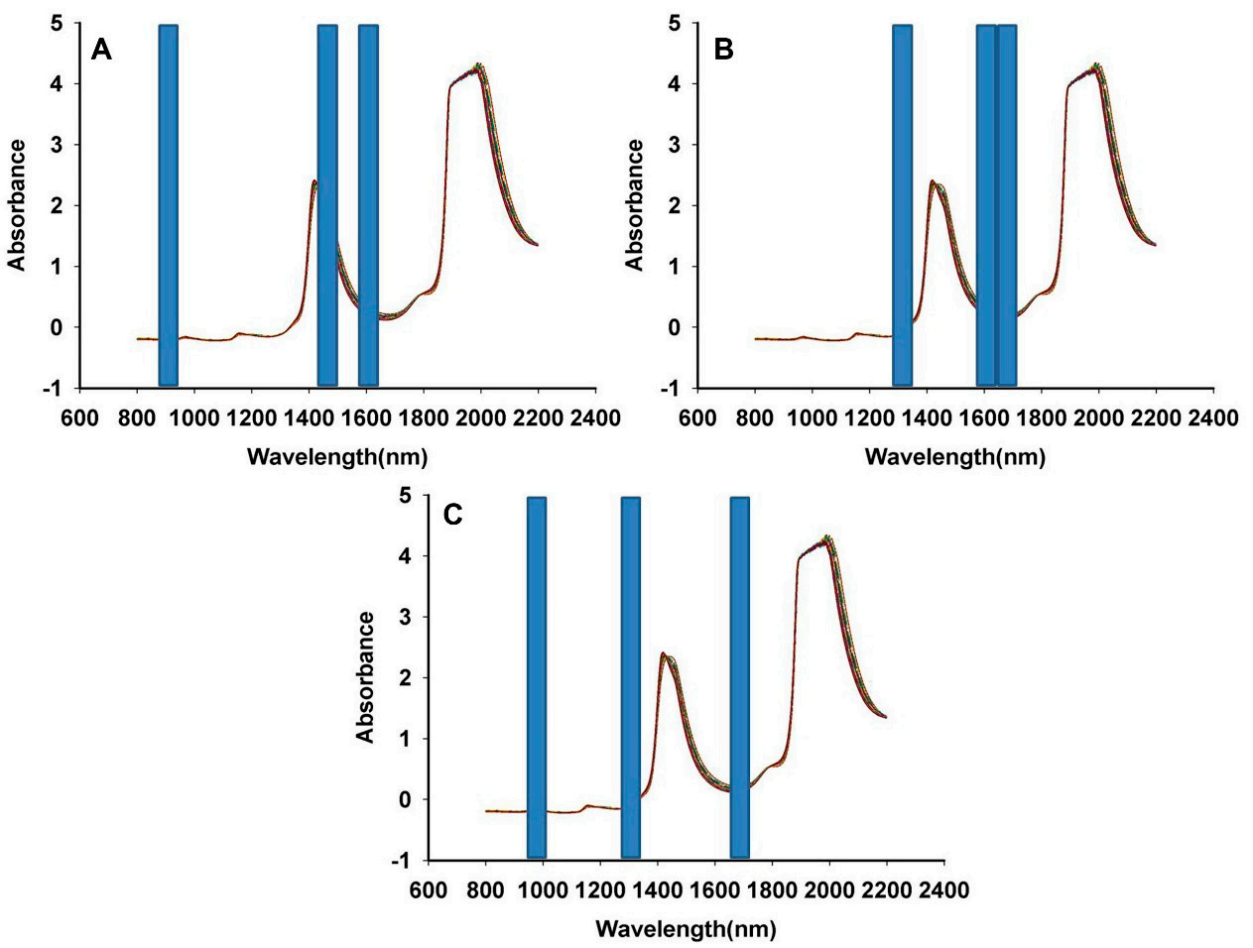
The optimum subinterval combination selected by SiPLS for the quantitative determination of hesperidin (**A**); naringin (**B**); neohesperidin (**C**).

**Figure 6:**
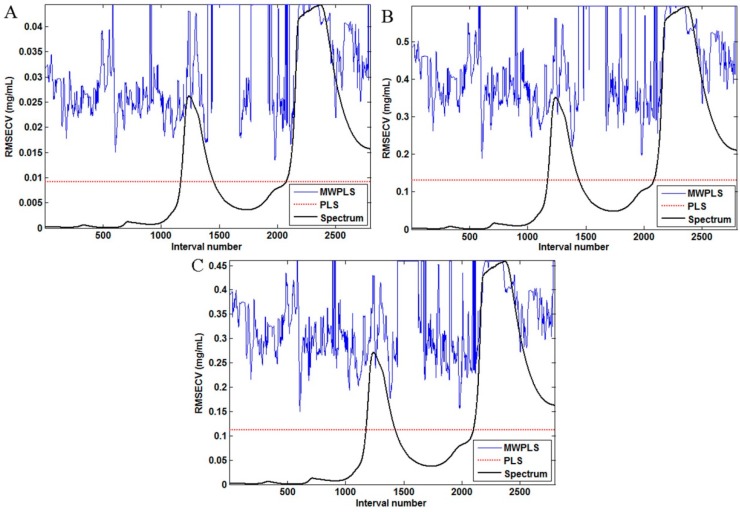
RMSECV for the best windows size for the full-spectrum models: hesperidin (**A**); naringin (**B**); neohesperidin (**C**) y-axis units for RMSEC and x-axis label and units (I believe these should be wavelength and nm, respectively.

Regarding hesperidin, for the SiPLS method, the optical subinterval combinations were 870–940 nm, 1430–1500 nm and 1570–1640 nm. Meanwhile, for naringin, the best combinations of interval wavenumbers were 1290–1360 nm, 1570–1640 nm and 1640–1710 nm. Finally, 940–1010 nm, 1290–1360 nm and 1640–1710 nm were selected using the optical SiPLS model of the neohesperidin, as described in the three blue regions in Figure 5. A MWPLS model was established for the three compounds in *Fructus aurantii*. It ranged from 800 to 2200 nm, and there were 2800 variables. There were between 13 and 41 moving windows of size *H*. As shown in Figure 6, all the RMSECV values of hesperidin, naringin and neohesperidin were higher than those of the full-spectrum PLS model, which showed that these three ingredients were not appropriate for use in MWPLS models.

### 3.5. Comparison of the Model Results by SiPLS and PLS Methods

**Figure 7:**
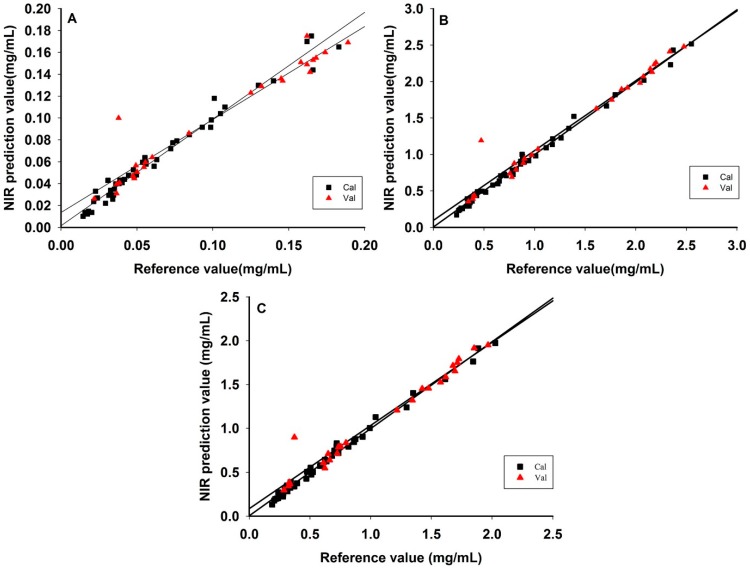
NIR predictions *versus* the reference method: hesperidin (**A**); naringin (**B**); neohesperidin (**C**).

According to the PLS and SiPLS methods the results for hesperidin, naringin and neohesperidin showed that the RMSEC, RMSECV, and RMSEP values and corresponding R^2^ produced using SiPLS were better than those produced using the PLS model. For this reason, SiPLS was used to establish the models for extraction process of *Fructus aurantii* (Table 4). Figure 7 shows the regression of calibration and prediction results for each SiPLS model. The results showed that the reference and prediction values were almost perfectly aligned in a straight line.

**Table 4:** The results of PLS and SiPLS model of three compounds.

Quality Parameter	Model	Calibration Set			Validation Set		Prediction Set	
		RMSEC *		R^2^	RMSECV *	R^2^	RMSEP *	R^2^
Hesperidin	PLS	0.0067	0.9760		0.0096	0.9530	0.0158	0.9237
	SiPLS	0.0066	0.9770		0.0079	0.9687	0.0155	0.9261
Naringin	PLS	0.0892	0.9776		0.1334	0.9499	0.1551	0.9544
	SiPLS	0.0469	0.9953		0.0550	0.9915	0.1493	0.9577
Neohesperidin	PLS	0.0774	0.9705		0.1108	0.9420	0.1197	0.9545
	SiPLS	0.0406	0.9919		0.0502	0.9881	0.1122	0.9599

* The unit of RMSEC, RMSECV and RMSEP was mg/mL.

### 3.6. Comparison of the Model Results by PLS Methods and Bagging-PLS

**Figure 8:**
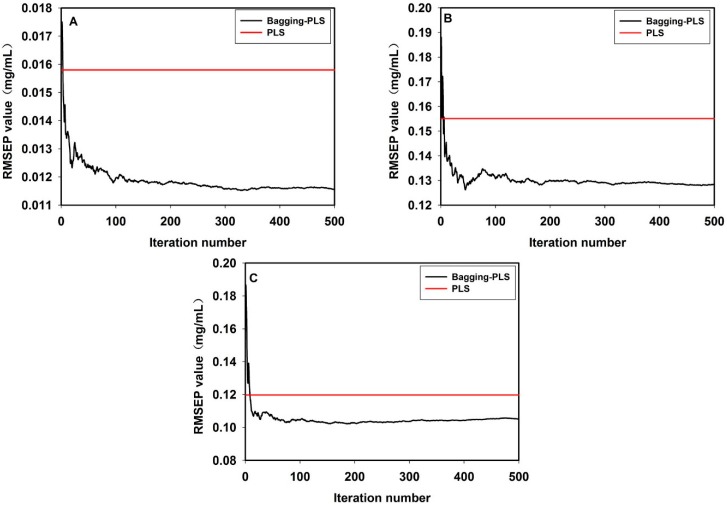
The full spectrum bagging-PLS *versus* full spectrum PLS: hesperidin (**A**); naringin (**B**), neohesperidin (**C**).

**Figure 9:**
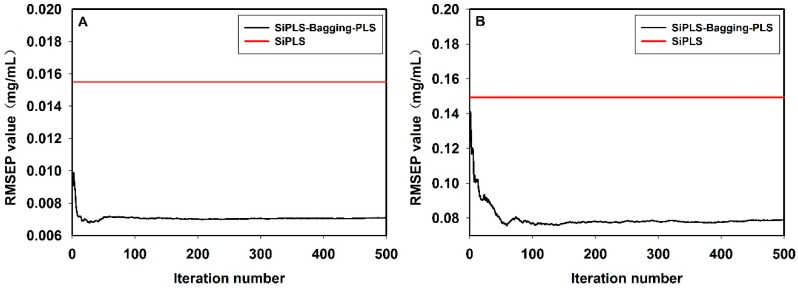

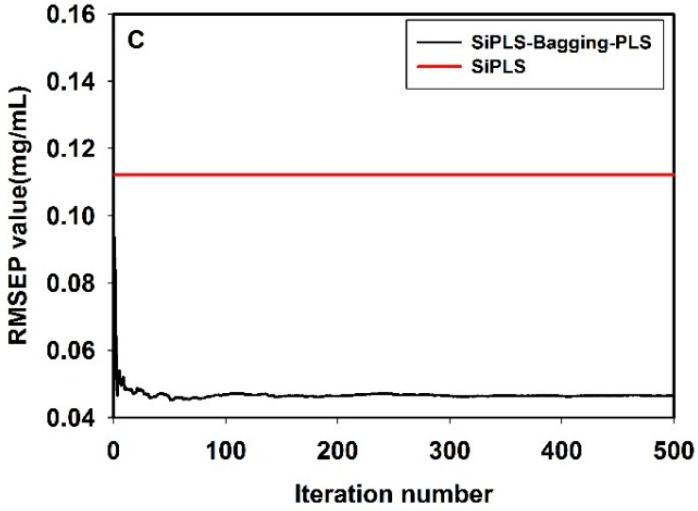
The SiPLS-bagging-PLS *versus* SiPLS: hesperidin (**A**); naringin (**B**); neohesperidin (**C**) Please add y-axis units.

In this work, the bagging-PLS method mentioned above was used to compare to the full spectrum PLS, meanwhile, SiPLS combined with bagging-PLS was used to compare to the SiPLS. As shown in Figure 8, firstly, for bagging-PLS, with the iteration number increasing from 0 to 500, the RMSEP value was gradually stabilized. The RMSEP values of the hesperidin, naringin and neohesperidin were 0.012, 0.13, and 0.105 mg/mL, respectively. Secondly, for the full spectrum PLS, the RMSEP values of the hesperidin, naringin and neohesperidin were 0.0158, 0.1551, and 0.1197 mg/mL, respectively. The results showed that all the RMSEP values of bagging-PLS using ensemble method, to the hesperidin, naringin and neohesperidin, were lower than those of the full-spectrum PLS model. Figure 9 shows the results of bagging-PLS, which were identical to those of PLS, for SiPLS coupled with bagging-PLS, the RMSEP values of the hesperidin, naringin and neohesperidin were 0.0071, 0.078, and 0.047 mg/mL, respectively. For SiPLS, the RMSEP values of hesperidin, naringin and neohesperidin were 0.0155, 0.1493, and 0.1122 mg/mL, respectively. All the results showed in Figure 8 and Figure 9 demonstrated that bagging-PLS was more robust than PLS alone and bagging-PLS was found to render more accurate predictions.

## 4. Conclusions

Throughout these experiments, bagging-PLS was proved to be more robust than PLS alone, which was found to improve prediction accuracy. Bagging-PLS can be used to construct models online using NIR sensors. The variable selection method SiPLS can improve the predictive accuracy and robustness of the models of hesperidin, naringin and neohesperidin, which demonstrated that SiPLS combined with bagging-PLS can improve the robustness of the models considerably. Bagging-PLS and NIR sensors are here put forward for use in the online quantitative monitoring of pilot-scale extraction processes in *Fructus aurantii*, and a strategy for online NIR control and monitoring of CHM is presented.
